# Recent Advancements in Chitosan-Based Biomaterials for Wound Healing

**DOI:** 10.3390/jfb16020045

**Published:** 2025-01-30

**Authors:** Jahnavi Shah, Dhruv Patel, Dnyaneshwari Rananavare, Dev Hudson, Maxwell Tran, Rene Schloss, Noshir Langrana, Francois Berthiaume, Suneel Kumar

**Affiliations:** Department of Biomedical Engineering, Rutgers, The State University of New Jersey, Piscataway, NJ 08854, USA; js3021@scarletmail.rutgers.edu (J.S.); dp1016@scarletmail.rutgers.edu (D.P.); dpr90@scarletmail.rutgers.edu (D.R.); db1247@scarletmail.rutgers.edu (D.H.); schloss@soe.rutgers.edu (R.S.); fberthia@soe.rutgers.edu (F.B.)

**Keywords:** chitosan, polymer complexes, wound dressing, wound healing, drug delivery

## Abstract

Chitosan is a positively charged natural polymer with several properties conducive to wound-healing applications, such as biodegradability, structural integrity, hydrophilicity, adhesiveness to tissue, and bacteriostatic potential. Along with other mechanical properties, some of the properties discussed in this review are antibacterial properties, mucoadhesive properties, biocompatibility, high fluid absorption capacity, and anti-inflammatory response. Chitosan forms stable complexes with oppositely charged polymers, arising from electrostatic interactions between (+) amino groups of chitosan and (−) groups of other polymers. These polyelectrolyte complexes (PECs) can be manufactured using various materials and methods, which brings a diversity of formulations and properties that can be optimized for specific wound healing as well as other applications. For example, chitosan-based PEC can be made into dressings/films, hydrogels, and membranes. There are various pros and cons associated with manufacturing the dressings; for instance, a layer-by-layer casting technique can optimize the nanoparticle release and affect the mechanical strength due to the formation of a heterostructure. Furthermore, chitosan’s molecular weight and degree of deacetylation, as well as the nature of the negatively charged biomaterial with which it is cross-linked, are major factors that govern the mechanical properties and biodegradation kinetics of the PEC dressing. The use of chitosan in wound care products is forecasted to drive the growth of the global chitosan market, which is expected to increase by approximately 14.3% within the next decade. This growth is driven by products such as chitoderm-containing ointments, which provide scaffolding for skin cell regeneration. Despite significant advancements, there remains a critical gap in translating chitosan-based biomaterials from research to clinical applications.

## 1. Introduction

Chitosan is a polymer that has emerged as a subject of scientific interest in the realm of wound healing. Chitin, the second-most abundant polymer usually found in the exoskeletons of crustaceans, undergoes a transformative deacetylation process to attain chitosan, which endows it with numerous unique and valuable properties. The basic structural unit of chitosan consists of two sugar molecules, N-acetylglucosamine (GlcNAc) and glucosamine (GlcN), connected through β (1–4) linkage [1,2]. The structural unit gives chitosan its unique properties of biodegradability, structural integrity, hydrophilicity, gel-forming ability, and controlled drug release [3,4,5]. The presence of amino groups in its structure makes it an excellent candidate for drug delivery systems and wound dressings [3] by forming electrostatic interactions with negatively charged mucosal surfaces and allowing muco-adhesion to chitosan-based drug delivery carriers. Chitosan also possesses hemostatic properties, which are essential for its application in surgical procedures [6].

Beyond its hemostatic properties, chitosan can function as a scaffold for tissue regeneration, promoting the formation of granulation tissue and aiding the natural healing process. Its antibacterial properties, coupled with its facilitation of the body’s defense mechanism, make it an excellent choice for preventing wound infections. Additionally, chitosan can be tailored to release bioactive agents, allowing for a customized approach to distinct types of wounds. Its applicability to films, hydrogels, and dressings makes it versatile in its wound-healing approach [5,6,7,8,9]. Depending on their source, chitosan molecules can exhibit variations in their size, degree of deacetylation, molecular weight, and crystallinity. A higher degree of deacetylation corresponds to a greater number of GlcN units, and this variability in deacetylation degree can impact the solubility, biocompatibility, and charge density of chitosan, ultimately affecting its potential in biomedical applications. Molecular weight is another notable variable that can alter viscosity, strength, mechanical properties, and drug delivery capabilities. Additionally, chitosan can exist in both amorphous and crystalline forms, thereby affecting its barrier properties and mechanical strength [10].

Chitosan plays a crucial role in the formation of polyelectrolyte complexes (PECs). These complexes arise from electrostatic interactions between the positively charged amino groups of chitosan and the negatively charged groups of other polymers [11] [12]. Among the most frequently utilized polymers in conjunction with chitosan are polygalacturonic acid (PgA), heparin sodium (HS), gum arabic (GA), sodium alginate (SA), and poly(acrylic acid) (PAA) [12,13,14,15,16,17]. These complexes typically form gel-like or film-like structures resulting from electrostatic forces. Their interactions ensure higher biocompatibility by balancing the charges and reducing surface charge density compared to individual polymers, thereby mitigating irritation and adverse reactions that may occur when individual polymers are used. By leveraging their effective delivery properties, PECs can incorporate nanoparticles, growth factors, or antimicrobial agents for enhanced wound-healing capacity [18,19,20]. A comprehensive literature review on PEC reveals significant variability in the manufacturing processes of products available in the market, such as hydrogels, films, dressings, and membranes (Table 1). These products cater to diverse biomedical applications, including wound dressings, nanodevices for skin lesions, drug-delivery systems, and nanoparticle-induced therapeutics for in vivo studies. This variability reflects the complex nature of products made using chitosan as the primary biopolymer, which helps achieve desired results like improved muco-adhesivity, antibacterial properties, controlled release, mechanical strength, and faster oxygen transmission rates. The table below introduces the variabilities observed in the types of chitosan used, including their degrees of deacetylation, molecular weights, sourcing, product thickness, and the temperatures used in their drying techniques. These differences help achieve desirable characteristics in the resulting films/dressings, hydrogels, and membranes. Although these variabilities can be advantageous, there is a need for uniformity in the manufacturing process and definitive requirements for the type of chitosan to be used and other preparation techniques to ensure the desired functionality in the products manufactured.

PEC films are a promising class of biomaterials with remarkable potential for wound healing [8,12,13]. They serve not only as protective barriers but also offer controlled drug delivery capabilities, enhancing the regenerative process of damaged tissue. This review article highlights the many properties of chitosan-based biomaterials, their impact on the wound-healing process, and the diversity in materials and methods employed in their construction. The impact of manufacturing methods and composition on film structure and biological response emphasizes the complexity inherent in optimizing PEC films for wound healing applications. These films function not only as passive dressings but also actively engage in the regeneration process by creating a microenvironment conducive to cell proliferation, adhesion, and migration—essential steps for tissue regeneration. Through alterations in porosity, thickness, and materials, they can be optimized to mimic the extracellular matrix of the wound site. Moreover, the incorporation of antibacterial nanoparticles, growth factors, and drugs further enhances their potential for effective wound management [11].

This review advances the field by providing chitosan-centric wound healing analysis and its compatibility with several polymers. Unlike prior studies that broadly cover PEC films, we focus specifically on the contributions of chitosan to the mechanical, biochemical, and biological properties of films, echoing its influence on wound healing efficacy. Furthermore, we also include a comprehensive list of multiple chitosan-based products on the market showcasing the feasibility of employing chitosan for wound treatment. For this review, extensive literature research was carried out through keywords such as chitosan wound dressing, PEC films, and chitosan topical drug delivery system via PubMed, Google Scholar, Journal of Mechanics, and Physics of Solids. Information was then narrowed down to chitosan-based biomaterials and related products for wound healing, with articles ranging from 2019 to 2025, and a consolidated database of approximately 80–90 articles was generated.

## 2. Chitosan Properties

### 2.1. Antibacterial Property

**Figure 1:**
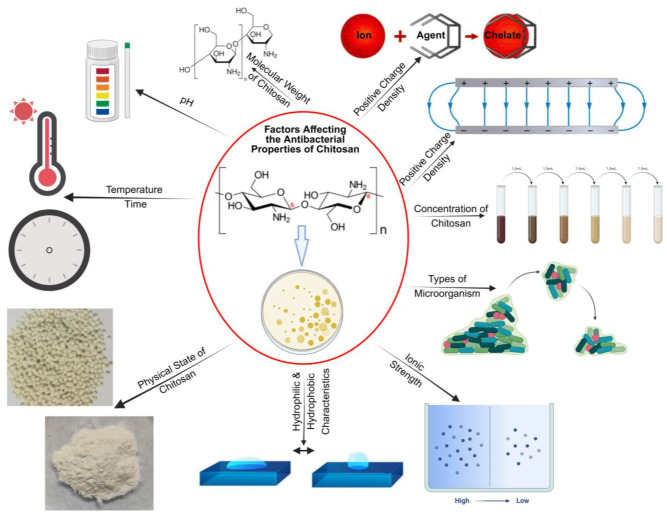
The figure depicts the different factors that affect the antibacterial properties of chitosan. Created with BioRender.com. Accessed on 25 November 2024.

Effective wound-healing biomaterials must possess antibacterial properties. While the antibacterial mechanism of chitosan is not fully understood, research has indicated several approaches that chitosan adopts towards this property. The simplest mechanism involves asparagine N-conjugated chitosan oligosaccharides possessing two positively charged sites and participating in electrostatic interactions with the negatively charged carboxyl groups on bacterial cell walls, altering the membrane permeability of bacterial cell walls and releasing intracellular materials. Another possible mechanism is that chitosan can function as a chelating agent, binding to trace metals and generating toxins that hinder bacterial growth. Chitosan typically has a pKa range of 5–6. When applied to a wound area of pH 5.5, the amino groups in chitosan become positively charged. This increased positive charge density further destabilizes the bacterial cell wall, thereby elevating the antibacterial effectiveness of chitosan [14,15,16,17,31]. The third mechanism is that chitosan diffuses through the cell membrane and adheres to bacterial DNA, disrupting its replication and hindering its growth at the wound site. In Gram-negative bacteria, chitosan interacts with anionic structures present on the surface, like lipopolysaccharides and proteins. In Gram-positive bacteria, however, it interacts with the cell wall layer consisting of negatively charged peptidoglycans and teichoic acids. The antimicrobial properties of chitosan are influenced (Figure 1) by several factors [31].

### 2.2. Mucoadhesive Property

As a result of its cationic character, chitosan exhibits mucoadhesive properties in its swollen states. This mucoadhesive attribute arises from non-covalent interactions such as hydrogen and ionic bonds between chitosan and mucin. The amino and hydroxyl groups form hydrogen bonds and ionic interactions with negatively charged mucosal surfaces, providing initial attachment. Then, the long polymer chains of chitosan form a network of interactions, like Van der Waals, hydrophobic interactions, etc., with mucin, thereby forming a cohesive gel-like layer that enhances the bioavailability of incorporated drug in chitosan-based dressing. Gradually, the chitosan penetrates the mucus layer and reaches the epithelial cells where it delivers the drug [19,20].

### 2.3. High Fluid Absorption Capacity

Chitosan’s hydrophilicity is attributed to the presence of –OH functional groups, which allow for water diffusion. This property is advantageous for effective drug delivery as water uptake is crucial. However, increasing film chitosan concentration does not necessarily enhance fluid absorption capacity. The viscosity increment associated with higher chitosan concentration impacts the film’s absorption capacity. Moreover, chitosan is important in absorbing wound exudate, aiding in maintaining a moist wound environment. This absorption leads to the gel formation of chitosan, where the gel-like matrix helps retain absorbed fluids, preventing leakage and patient discomfort. Chitosan’s versatility in absorbing various fluids such as water, blood, and wound exudates makes it suitable for treating different types of wounds. Efficient absorption also helps prevent secondary infections by minimizing bacterial proliferation in drier environments [35,36,37,38,39,40,41].

### 2.4. Biocompatibility

Chitosan films display high biocompatibility. It was verified by observing that experimental mouse embryonic fibroblast cells, cultured in chitosan-bound microtubes in media, proliferated at the same rate as control cells that only contained water in the media. Additionally, zwitterionic chitosan, which is soluble in water at pH levels below its isoelectric point (pI), demonstrates biocompatibility with blood components and shows toleration upon intraperitoneal injection. Chitosan shares structural similarities with glycosaminoglycans found in natural tissues. Upon enzymatic hydrolysis, chitosan degrades into non-toxic byproducts such as glucosamine. These byproducts occur naturally in the body and are generally compatible, further supporting the biocompatibility of chitosan for medical applications [31,42,43].

### 2.5. Biodegradability

Chitosan is biodegradable due to the presence of hydrolyzable glycosidic bonds, such as -GlcN-GlcN-, -GlcN-GlcNAc-, -GlcNAc-GlcN-, and -GlcNAc-GlcNAc- linkages. These linkages are easily hydrolyzed by human enzymes found in lysosomes. Chitosan films represent biocompatible materials that gradually break down into harmless products that are absorbed by the body. This slow breakdown mechanism ensures that the film does not degrade prematurely before the wound heals, thus extending the therapeutic window for a complete wound [8,42,44].

### 2.6. Anti-Inflammatory Response

Chitosan film triggers an anti-inflammatory response upon topical application to open wounds. Its anti-inflammatory effects make these films advantageous for treating prolonged inflammation at wound sites [8]. For example, chitosan suppresses the secretion and expression of proinflammatory cytokines and nitric acid synthase in astrocytes, which actively participate in cytokine-mediated inflammatory responses. Furthermore, it exerts anti-inflammatory effects by inhibiting prostaglandin E2 and cyclooxygenase-2 protein expression, consequently reducing proinflammatory cytokines such as tumor necrosis factor-α and interleukin-1β. Additionally, chito-oligosaccharides display superior anti-inflammatory properties compared to the nonsteroidal anti-inflammatory drug indomethacin. Chitosan formulated for wound management may also induce analgesia by delivering a cool, pleasant, and soothing effect when applied to open wounds [44,45].

### 2.7. Antioxidant Property

Chitosan contains amino and hydroxyl functional groups that are capable of donating electrons. These groups scavenge free radicals like superoxide anions, hydroxyl radicals, and hydrogen peroxide and neutralize them, thereby mitigating cellular damage caused by these free radicals. Transition metal ions like iron, copper, etc., can catalyze the generation of reactive oxygen species (ROS), through Fenton and Haber–Weiss reactions. The chelating property of chitosan can make these metal ions unavailable, thereby suppressing their role in oxidative damage [44,46].

## 3. Types of Chitosan and PEC Films

**Figure 2:**
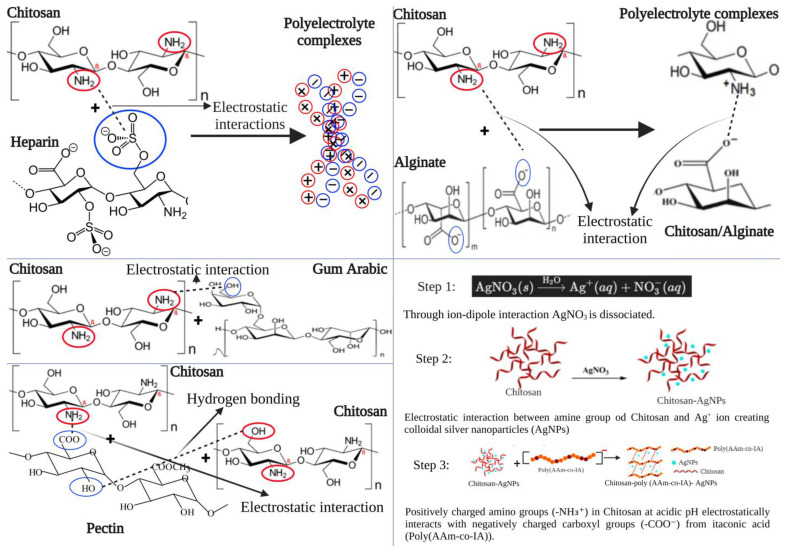
Electrostatic and hydrogen bonding interactions between chitosan and other polymers. Red color circles represent amino group (+) of chitosan and blue color circles represent different negatively charged groups. Created with BioRender.com. Accessed on 9 January 2025.

Chitosan’s biodegradability and biocompatibility make it a suitable material for the wound-healing process. However, biopolymers like chitosan have poor mechanical stability, which is why they are often used in conjunction with other polymers that can overcome this drawback. Chitosan can be paired with a variety of oppositely charged compounds to form PEC (Figure 2).

### 3.1. Chitosan–Heparin Sodium (CS-HS) Hydrogels

Chitosan (CS) has previously been utilized in combination with heparin sodium (HS) to create hydrogels. CS-HS hydrogel films, with ratios of CS and HS ranging from 1:1.2 to 1:0.5, self-assemble through hydrogen bonding and electrostatic interactions [18,47,48]. Together, they have greater mechanical performance than simple CS hydrogels because of the electrostatic interactions between CS and HS that enhance the mechanical properties. Research conducted by Shu et al. demonstrated that films with the lowest HS content exhibited the poorest performance in stress and strain tests, indicating that hydrogen bonds formed between CS molecular chains alone are unable to withstand large stresses. CS-HS hydrogel films can have their charges facially altered by adjusting the pH of the soaking solution, ensuring its adhesiveness and enabling it to withstand a weight of 20 g when adhered to the surface of human skin. This adhesiveness, dependent on pH, is quite useful; when applied to the wound, the hydrogel will adhere properly due to the acidic pH of the wound. As the wound heals, the pH will begin to neutralize, and the film will start to detach without causing much pain [18]. CS-HS hydrogels can significantly enhance wound healing due to the blood coagulation properties of CS and the immune cell migration facilitated by HS. During the inflammatory stage of wound healing, CS promotes the infiltration and migration of inflammatory cells. However, as the HS and macrophage interactions compete with the CS-HS interactions, the HS component is released, promoting blood circulation and reducing the formation of thrombus. The positively charged chemokines aggregated at the wound site are absorbed by the negatively charged HS chains, which helps to reduce the inflammatory response at the wound site and accelerate the overall healing process [18].

### 3.2. Chitosan–Sodium Alginate (CS-SA) Hydrogels

Additionally, chitosan can be combined with sodium alginate (SA) to form CS-SA hydrogels that are extensively studied for their potential in wound healing applications [49,50]. The electrostatic interactions between the positively charged amino groups of chitosan and the negatively charged carboxylate groups of SA form a stable complex. These hydrogels exhibit essential swelling properties that maintain a moist wound environment, which is crucial for the healing process. The swelling results from interactions between the CS-SA polymer chains and water molecules, while cross-linking protects the polymer from dissolution.

The CS-SA hydrogels also mimic the native extracellular matrix, providing a three-dimensional structure that supports cell proliferation and migration [51,52]. Different CS-SA ratios, such as 30/70 (CS-SA30), 35/65 (CS-SA35), 40/60 (CS-SA40), 45/55 (CS-SA45), and 50/50 (CS-SA50), have been explored to optimize their properties. As the alginate concentration increases, the hydrogel’s ability to absorb water and swell also increases. Among these combinations, the CS-SA30 formulation exhibits the highest water absorption, while Chi-SA50 demonstrates the lowest. Additionally, CS-SA50 has been shown to promote the migration of primary mixed neuronal cells (PMNCs) after 5 days in culture during a migration and adhesion study, indicating its potential in tissue engineering applications [51,53]. Further studies have investigated ratios such as 60/40 (CS-SA60), 80/20 (CS-SA80), and 90/10 (CS-SA90). The CS-SA60 hydrogel displays the highest elastic modulus under physiological conditions, whereas CS-SA90 shows the lowest, with no long-distance electrostatic interactions.

### 3.3. Chitosan–Gum Arabic (CS-GA) Films

Chitosan–gum arabic (CS-GA) films have also been studied [21]. Dispersions of low-molecular-weight CS-GA (LCS-GA) and high-molecular-weight CS-GA (HCS-GA) were prepared with varying pH values and mixing ratios. Positive zeta potential indicated the successful interaction of CS and GA, forming globular microstructures due to the branched structure of GA and the long-chain structure of CS. These oppositely charged biopolymers created complex structures based on network formation and various sites for trapping water. This quality demonstrates the high water uptake capacity of the film, essential for drug delivery applications. However, excessive water absorption can lead to matrix deformation as a result of chain reorientation and shrinkage. At an LCS-GA ratio of 1:0.75 and HCS-GA ratio of 1:1, an increase in GA amount resulted in higher CS tensile strength, indicating enhanced film strength compared to films with only CS. This suggests that increasing the GA amount to an optimal range can strengthen the film. The antioxidant, antimicrobial, and mucoadhesive properties were also improved with the same optimal ratio due to the greater flexibility of the biopolymer backbones [21,54,55].

### 3.4. Chitosan–Pectin (CS-P) Films

Further research has shown that chitosan can be combined with pectin (P) in different CS-P weight ratios (1:1, 1:2, 1:3, 1:4, 1:6, and 1:10) to produce periodontal films. These films are formed in an acidic pH environment. The electrostatic interactions between the amino groups of chitosan molecules and negatively charged carboxylate groups of pectin are responsible for the formation of these complexes. As the amount of pectin increases, the optimum polymer interactions also increase due to the presence of a sufficient number of ionized carboxylic acid groups [34,56,57,58,59]. Additionally, there is another component of pectin called polygalacturonic acid (PgA), which is widely used conjointly with chitosan to form PECs for bone tissue engineering, surgical adhesion, and wound healing [12,13,53,60,61,62,63,64,65]. However, this complex is not yet widely used. Recently, our lab manufactured a chitosan–PgA dressing, which was found to accelerate wound healing in diabetic mice induced with full-thickness skin wounds. In addition, it promoted hair growth around the scar area in the mice, surpassing the results observed with other vehicle controls [12,13]. We also discovered that it serves as an effective slow drug delivery vehicle [13]. PgA was introduced into the film to improve the dressing’s mechanical characteristics to maintain the integrity of the film through long-term use. However, PgA has also been suggested for its antimicrobial and immunomodulatory properties when combined with other polymers [66]. Therefore, the chitosan–PgA dressing is highly recommended for chronic wound healing.

### 3.5. Chitosan–Poly (AAm-co-IA)-AgNO_3_ IPC Films

Studies have demonstrated the formation of an inter-polyelectrolyte complex (IPC) film from chitosan, poly (AAm-co-IA), and AgNO_3_ nanoparticles. Chitosan reduces silver ions into colloidal silver to form a Chi/AgNPs solution. When this solution is mixed with poly (AAm-co-IA), electrostatic interactions between oppositely charged macromolecular chains occur, leading to PEC film production. These films exhibit antibacterial properties, as evidenced by a ‘zone of inhibition’ with a diameter of 2.6 cm in a Petri dish. In contrast, when only a Chi/poly (AAm-co-IA) film is used, the zone of inhibition has a diameter of 1.5 cm [22,31].

## 4. Chitosan-Based Innovations in Drug Delivery Systems

A complex structure of interconnected polymeric chains, presented as a hydrogel, has previously been shown to augment the proliferation of cells in vitro and enhance wound healing efficiency by accelerating the wound closure rate [67]. These hydrogels, formed through the cross-linking of chitosan and alginate, provide substantial support for cell growth [68,69,70]. Chitosan’s properties—including high biocompatibility, excellent biodegradability, low toxicity, and widespread availability at a low production cost—have made it a popular choice in the development of drug delivery systems [71,72]. For instance, a pH-sensitive hydrogel system, consisting of ethyl chitosan and alginate blended with genipin, a naturally occurring protein cross-linker, was developed to regulate protein drug delivery. This system acts as a polymer carrier for site-specific protein drug delivery within the intestine [73,74]. Similarly, a pH-sensitive CS-SA hydrogel embedded with microparticles was designed to deliver the antituberculosis drug rifampicin [75,76].

In the wound-healing domain, chitosan-based PEC systems have demonstrated significant advantages over conventional materials. Wound dressings play a vital role in facilitating healing, yet many existing dressings face limitations, such as rigidity, a lack of porosity, low mechanical strength, a tendency to adhere to the wound surface, and limited antimicrobial activity. To address these issues, a porous three-dimensional film was developed using a chitosan/carboxymethyl pullulan PEC loaded with 45S5 bioglass (CCMPBG). These films, fabricated via the freeze-drying method, offer enhanced mechanical strength, a controlled rate of swelling, and biodegradation behaviors, compared to the control chitosan/carboxymethyl pullulan (CCMP) film. These improvements are attributed to the interaction between the polymer matrix and the 45S5 bioglass. Compared to control films, the CCMPBG films demonstrated superior biocompatibility, antimicrobial activity, and wound closure ability due to the combined effects of chitosan, carboxymethyl pullulan (CMP), and bioglass (BG). The findings suggest that CCMPBG films can serve as effective dressing materials for wound therapy [29].

Further advancements have been made in incorporating bioactive agents into chitosan-based wound dressings. For example, chitosan–alginate films comprising poly(dimethylsiloxane) loaded with thymol and beta-carotene were designed to improve wound healing by leveraging antioxidant, anti-inflammatory, and anesthetic properties of these compounds. The formulation displays high stability when exposed to physiological fluids or conditions simulating patient bathing. The supercritical carbon dioxide impregnation/deposition method (conducted at 250 bar and 45 °C for 14 h, employing two depressurization rates of 5 and 10 bar/min) used to produce these films enabled the formation of a PEC facilitating favorable bioactive–polymer interactions, resulting in sustained release profiles during in vivo applications [77,78].

Multifunctional PEC hydrogels have also been explored as drug delivery platforms. For instance, the CS-HS hydrogel films presented by Shu et al. demonstrated high antibacterial efficacy against *Escherichia coli* at specific pH levels or in coordination with metal ions, significantly contributing to expedited wound healing. The self-assembly approach presented in this study may serve as a versatile strategy for fabricating multifunctional PEC hydrogels, catering to a wide array of biomedical applications [18]. The layer-by-layer deposition technique for the development of PEC films, initially introduced in the early 1990s, offers numerous advantages in biomedical applications. This methodology facilitates the diffusion of molecules into films, thereby regulating their internal structures and controlling their mechanical properties, enabling the design of biomedical devices that can encapsulate bioactive molecules for wound therapy and other applications. Recent advancements in this methodology have resulted in the development of new biomaterial coatings and tissue-engineered models that can mimic in vivo cellular microenvironments, including those supporting stem cells [44].

By incorporating advanced drug delivery systems into wound care, chitosan-based biomaterials offer inventive solutions in the development of multifunctional, therapeutic wound dressings. Such systems effectively address critical challenges such as infection control, mechanical stability, and the sustained release of bioactive agents, establishing wound-healing films as fundamental constituents of modern wound management approaches.

## 5. Wound Healing Products

Based on market demand, the global chitosan market is expected to see approximately a 14.3% increase within the next decade. This growth is driven by demand in several countries, including the United Kingdom, China, Iceland, and the United States of America [79]. In today’s market, medical devices and cosmetic products containing chitosan are available in different bandages and skincare ointments. Though the specific percent formulation of chitosan in these products is limited, the emphasis on chitosan in advertisements by these companies suggests that the percent composition is likely on a higher scale. For instance, companies such as Marine Polymer Technologies, Tricol Biomedical Inc. (HemCon), and Celox use chitosan [80] in their bandage/gauze drug delivery systems (Table 2). These products are designed to aid first responders, military personnel, domestic law enforcement, and the general public. They are commonly used in emergency and surgical settings to control severe bleeding from hemodialysis at percutaneous needle access, vascular access, and percutaneous catheter access sites. Additionally, they offer an antibacterial barrier against a wide range of Gram-positive and Gram-negative organisms, including antibiotic-resistant *Staphylococcus aureus* (MRSA), *Enterococcus faecalis* (VRE), and *Acinetobacter baumannii* [81,82].

Moreover, in the beauty and cosmetic field, chitosan can be found in many ointments and skincare products. A company named 47Skin has developed a product that combines silver and chitosan, called chitoderm (Table 2). The integration of silver creates an antibacterial shield over the skin. The structure of chitoderm utilizes a lattice-like arrangement, which encourages skin cells to regenerate. This combination reduces the appearance of hyperpigmentation and scarring by decreasing melanin production. Furthermore, chitosan promotes the shedding of older skin cells and the production of newer ones at a faster rate. Although the exact percentage of chitosan in these products is not known, it is assumed to work synergistically, with chitosan being the majority component and silver performing similarly to trapped metal ions for antimicrobial purposes [86]. There are several patents filed on chitosan-based production and their application in wound healing and related problems as described in Table 3.

## 6. Research Gaps

While chitosan’s properties and its potential in wound healing have been studied extensively, several significant research gaps are necessary to address and fully utilize its capabilities. These gaps not only limit the current application of chitosan-based biomaterials but also highlight opportunities for future innovation in the field of wound healing.

### 6.1. Standardization Challenges and Manufacturing Variability

One of the prime challenges in chitosan research is the lack of standardization in its preparation and characterization. Variability in molecular weight, the degree of deacetylation, and source material (e.g., shrimp shells or plant sources) can lead to inconsistent mechanical properties, biodegradability, and biological activity. Standardized protocols for producing and characterizing chitosan are essential to ensure uniformity in both research studies and clinical applications [1,2,3].

PECs featuring chitosan show considerable promise, particularly as wound dressings. However, there is a lack of consistency in manufacturing techniques, such as layer-by-layer casting, freeze-drying, and electrospinning (Table 1), which produces much variability in the resulting film thickness, porosity, and drug release profiles. Therefore, developing scalable and reproducible manufacturing methods to optimize the therapeutic effects of chitosan-based biomaterials is critical for clinical adoption [18,57].

### 6.2. Limited Clinical Translation

Although numerous in vitro and in vivo studies have demonstrated the efficacy of chitosan-based treatments for healing, their translation to clinical settings remains limited. Large-scale clinical trials are required to validate the safety and success of these materials across diverse wound types and patient populations. Additionally, the regulatory landscape for chitosan-based products is still evolving, creating hurdles for commercialization [8,12,44].

### 6.3. Long-Term Biocompatibility and Biodegradability

The long-term biocompatibility and biodegradability of chitosan-based materials in human applications are not fully understood. While chitosan’s enzymatic degradation produces non-toxic byproducts, more research is needed to assess the potential for adverse reactions, especially in chronic wound settings or prolonged applications [68,69].

### 6.4. Integration with Existing and Emerging Therapies

Chitosan-based biomaterials possess the potential to be combined with existing and emerging therapies, such as growth factors, stem cells, and bioactive nanoparticles. However, in order to optimize such integrations, such as applying sustained release mechanisms, further investigation is required [86,88].

### 6.5. Environmental and Economic Considerations

The sourcing and production of chitosan raises concerns regarding its environmental and economic effects. The reliance on sourcing the compound from crustacean shells can be a barrier to sustainable production, and alternative sources remain uncharted. Additionally, cost-effective manufacturing processes need to be developed to make chitosan-based products accessible for widespread use [81,88].

## 7. Conclusions

Chitosan-based biomaterials hold tremendous potential in the revolutionary area of wound healing and tissue regeneration due to their intrinsic features, such as biocompatibility, biodegradability, and multifunctionality. Such characteristics of chitosan give it the potential to act as a versatile substrate for a wide range of applications, including in wound dressings, hydrogels, membranes, films, and drug delivery systems. Chitosan has been used for applications in supporting cellular activities, such as adhesion, proliferation, and migration, all favorable aspects of creating microenvironments conducive to healing. While the benefits of chitosan are prevalent, there continue to remain a lot of challenges in using chitosan for wound management. Variability in molecular weight, the degree of deacetylation, and source may cause significant differences in its mechanical properties and biological efficacies.

Standardizing these parameters is essential to ensure consistent outcomes across applications. This review offers an in-depth analysis of the natural features of chitosan and related products in combination with other polymers for its application in wound healing and tissue regeneration. This article emphasizes the broad versatility of chitosan by covering its role in forming biomaterials for use in wound healing applications and its potential integration with emerging technologies like nanoparticles and growth factors. In addition, this review pinpoints significant knowledge gaps, such as standardization/manufacturing variability issues and the challenges in translation to the clinic, with concrete strategies to overcome them. Furthermore, translating laboratory results into clinical practice demands addressing scalability, cost-effectiveness, and regulatory barriers. The high cost of large-scale clinical trials provides an obstacle to the generalized acceptance of chitosan-based biomaterials in healthcare. Another key priority in this regard will undoubtedly include ensuring sustainability in sourcing chitosan, and filling in such gaps is expected in advancing the field of chitosan-based biomaterials. Future studies should be channeled toward standardization in production methods, conducting large-scale clinical trials and exploring innovative combinatorial approaches with various advanced therapies. Overcoming these challenges will ensure that chitosan-based products transition from a promising biopolymer in research settings to reaching wound care clinics to benefit patients and the healthcare system.

## Figures and Tables

**Table 1:** Overview of variability in chitosan-based matrix manufacturing.

Product Types	Types of Chitosan Used			Manufacturing Variability in the Casting/Drying Process	Thickness	Advantages	Area of Improvement for Clinical Use	Reference
	Degrees of Deacetylation	Molecular Weights	Source(s)					
▪ Films ▪ Dressing ▪ Hydrogel ▪ Membrane	75–96%	15–700 kDa (low and medium MW)	▪ Shrimp shells (animal source) ▪ Plant-based powder or extracts	Layer by layer: room temperature to 150°C drying. Self-assembly: hot plate/molds from 60 to 100 °C. Casting: drying temperature from 37 to 50 °C. Fabrication: freeze-dried to 60 °C using different techniques. Lyophilization: hot air and supercritical CO_2_ drying at constant 33 °C. Freeze drying: different techniques such as hot air, fused zirconia/alumina/silica/crucible, supercritical CO_2_ drying, NH3 evaporation methods, supercritical CO_2_ impregnation/deposition (SSI/D) method, and multilayering in a polyion solution.	300 nm to 220 µM	▪ Wound-healing promotor ▪ Some sort of transparency to observe the wound during the healing process ▪ Impermeable to bacteria and water ▪ Porosity allows the water vapor from beneath the dressing to the external environment transmission. ▪ Structure varying thickness ▪ Mechanical stability ▪ Antibacterial and biodegradability ▪ Tissue regenerative properties	▪ Critical variables such as temperature, composition, source, MW, and acetylation/deacetylation should be effectively regulated. ▪ The alternative of plasticizers. ▪ Transparency is unappealing in some cases. ▪ Packaging and delivery of wound-healing specific molecules. ▪ Suitable dressing for high exudative injuries. ▪ An adhesive dressing is needed to prevent leakage. ▪ Avoid frequent dressing. ▪ Suitable for all wound types including fragile skin. ▪ Non-painful soft dressing.	[11,12,18,21,22,23,24,25,26,27,28,29,30,31,32,33,34]

MW—molecular weight; RT—room temperature.

**Table 2:** Overview of chitosan-based marketed products.

Product Name	Company/ Founded	Product Type	Chitosan(%)	Deacetylation(D)/Acetylation (A) (MW)	Application	Country of Origin	FDA Approval (Year)	References
HemCon Bandages	Tricol Biomedical 2001	Dressing	90–95%	D—70–95% A-85–95% (100–500 kDa)	Prevent Bleeding Antibacterial	Oregon, USA	Yes (2003)	[80,81]
ChitoFlex Pro Hemostatic Dressing	Tricol Biomedical 2001	Dressing	~95%	D—70–95% A—85–95% (100–500 kDa)	Control Bleeding Antibacterial	Oregon, USA	Yes (2003)	[80,83]
Celox Gauze	Celox 2010	Gauze Dressing	10–30% by weight	D: 70–95% A: 5–30% (10–1000 kDa)	Emergency Bleeding Control	Crewe, UK	Yes (unclassified medical device)	[84,85]
Silver Chitoderm	47Skin 2018	Cosmetic Product	70–90%	D—70–95% A—5–30% (50–1000 kDa)	Scar Reduction	USA	Not subjected to premarket approval as it is cosmetic product	[86,87]

**Table 3:** Summary of patents related to chitosan-based products.

Serial #	Patent Number	Applications and Significant Features	Brief Product Description
1.	US-11160901-B2	Bioadhesive chitosan gel to control bleeding and promote healing with scar reduction without obscuring or interfering with access to a surgical field.	This is an aqueous chitosan gel system of novel non-scarring, non-interfering, transparent, stable, and solubilized chitosan, which is aimed at controlling bleeding. The gel system comprises water, chitosan, acid, a plasticizer, a rheology modifying agent, antioxidant stabilizing, alcohol, and multivalent salt. Furthermore, specific components within the chitosan gel system can comprise multiple types of organic acids, such as bifunctional/unfunctional/multifunctional acids, phosphoric acids, and salt.
2.	US-11718828-B2	Cartilage gel for cartilage repair, comprising chitosan and chondrocytes.	This invention concerns methods for obtaining an implantable cartilage gel for the tissue repair of hyaline cartilage, comprising chitosan hydrogel and cells that are capable of forming hyaline cartilage; said method comprises a step for the amplification of primary cells in a 3-D structure with a physical hydrogel of chitosan. Furthermore, an additional step for re-differentiation and induction for synthesizing the ECM through cells such as the articular chondrocytes and mesenchymal stem cells will be integrated for better repair properties.
3.	US-20230270914-A1	Designed for easy and effective application to control severe bleeding in various wound conditions. Composed of chitosan-based Celox particles and woven gauze substrate.	The invention relates to a hemostatic dressing that combines Celox, a chitosan-based material known for its blood-clotting properties, with a gauze substrate. This combination enhances the effectiveness of the dressing in controlling severe bleeding. The gauze provides a supportive matrix for the Celox particles, ensuring they remain in place and making the application easier and more efficient in emergencies.
4.	US4572906-A	Chitosan-based wound dressing materials.	This patent mentions an invention of a surgical dressing to help protect wounds during healing. The dressing proposes a proprietary blend of gelatin and chitosan in a weight ratio of about 3:1 to 1:3. Furthermore, it also suggests incorporating a plasticizer ranging from 0 to 40% *w/v* as per the combined weight of gelatin and chitosan.
5.	US9192574-B2	Chitosan paste wound dressing.	This patent proposes a method of treating a wound with a ready-to-use composition. The composition contains a high concentration of water-soluble chitosan in a phosphate-containing solution. It is a paste at typical room temperature, has a pH of at least 4, adheres to the body tissue/surgical site, and has a total residence time of 1 day.
6.	CN101816802-B	A hemostatic dressing mostly composed of chitosan and alginic acid.	This patent describes a medical dressing made from chitosan, a biocompatible and biodegradable polysaccharide derived from chitin, which is found in the exoskeleton of crustaceans. The dressing is designed for wound care, particularly to control bleeding and promote healing.
7.	CN107530470-B	Chitosan wound dressing comprises chitosan chitin, at least one triprotic acid, and at least one solubilizing acid.	This invention focuses on the development of a wound dressing that leverages the beneficial properties of chitosan, a biopolymer known for its biocompatibility, biodegradability, and antimicrobial properties. The dressing is designed to enhance wound healing by creating a moist environment, which is conducive to tissue repair and regeneration.

ECM—extracellular matrix; 3-D—3 dimensional.

## Data Availability

Not applicable.
